# The Neural Representational Space of Social Memory

**DOI:** 10.1162/opmi_a_00021

**Published:** 2019-02-01

**Authors:** Sarah L. Dziura, James C. Thompson

**Affiliations:** Department of Psychology, George Mason University, Fairfax, VA, 22030 USA; Department of Psychology, George Mason University, Fairfax, VA, 22030 USA

**Keywords:** social networks, social cognition, learning, representational similarity analysis, fMRI

## Abstract

Social functioning involves learning about the social networks in which we live and interact; knowing not just our friends, but also who is friends with our friends. This study utilized an incidental learning paradigm and representational similarity analysis (RSA), a functional MRI multivariate pattern analysis technique, to examine the relationship between learning social networks and the brain’s response to the faces within the networks. We found that accuracy of learning face pair relationships through observation is correlated with neural similarity patterns to those pairs in the left temporoparietal junction (TPJ), the left fusiform gyrus, and the subcallosal ventromedial prefrontal cortex (vmPFC), all areas previously implicated in social cognition. This model was also significant in portions of the cerebellum and thalamus. These results show that the similarity of neural patterns represent how accurately we understand the closeness of any two faces within a network. Our findings indicate that these areas of the brain not only process knowledge and understanding of others, but also support learning relations between individuals in groups.

## INTRODUCTION

Social relationships guide and support much of human behavior. Not only do we form strong lifelong family bonds, we also interact with others in work, education, and leisure settings and create lasting non-kin relationships. For many species, including humans, non-kin–based social networks can have important consequences for health and fitness (Cheney 2011; Tung et al., 2015). Benefits of social relationships can come both from direct connections (our friends) as well as second-order or indirect connections (friends of our friends) (Brent 2015; Seyfarth & Cheney, 2015). Knowledge of social ties involves a network of brain regions including bilateral posterior superior temporal sulcus (pSTS) and temporoparietal junction (TPJ) (Bault, Pelloux, Fahrenfort, Ridderinkhof, & van Winden, 2015; Satpute, Badre, & Ochsner, 2014). Learning and representing information about social hierarchies also recruits amygdala, hippocampus, and ventral mPFC (Kumaran, Melo, & Duzel, 2012; Zink et al., 2008). Recently, the position of individuals in one’s own social network were found to be represented in lateral temporal, inferior parietal, and medial and lateral prefrontal cortices as participants viewed videos of network members (Parkinson, Kleinbaum, & Wheatley, 2017). These findings suggest that information about personal social connections is represented in patterns of fMRI responses elicited by viewing the individuals from one’s real-world network. However, less is known about how we learn, remember, and represent information about new and indirect social connections. Understanding this process is important, as how we interact with our extended social network is often influenced by our knowledge of established relationships.

One of the ways that we acquire this knowledge is by observing patterns of associations between individuals (De Soto, 1960). The frequency of interaction between individuals can provide an important index of their relationship strength (Freeman, Freeman, & Michaelson, 1988; Lin, Dayton, & Greenwald, 1978). In nonhuman primates, observation of the frequency of association is suggested to be an essential cue to learning information about social connections (Seyfarth & Cheney, 2012; Tiddi, Sorrentino, Fischer, & Schino, 2017). Studies of the cognitive representation of social networks in humans have employed artificial social networks learned through paired associates learning-type paradigms (De Soto, 1960; De Soto & Bosley, 1962; Janicik & Larrick, 2005; Zitek & Tiedens, 2012). Artificial networks give the ability to experimentally control network statistics, for instance by varying the centralization (the extent to which a network is grouped around a particular point), which can affect the efficiency of information passed among members and consequently group performance (Leavitt, 1951). They also allow us to assess the role of the memory (in)accuracy for relationship characteristics in participants’ representations of social networks (Brashears, 2013; Zitek & Tiedens, 2012). Humans can learn quite complex statistical relationships between stimuli through simple associations, even in the absence of explicit knowledge of these relationships (Turk-Browne, Scholl, Johnson, & Chun, 2010). We expect that such associative learning plays an important role in acquiring knowledge about social connections. We also expect that the accuracy with which participants recall these connections plays a role in how these associations are represented neurally.

We examined the memory and neural representation of connections between members of two novel social networks of varying centralization, using fMRI and representational similarity analysis (RSA). We examined if the pattern similarity of fMRI responses to any two faces from a learned social network reflected the tie strength (closeness) of those two individuals within the network: that is, does the similarity of the pattern of response to two network members increase as a function of the closeness of those members? We also examined if the memory for tie strength between network members was related to the similarity of the fMRI voxel pattern response to the faces of members.

## MATERIALS AND METHODS

### Participants

Twenty-two healthy individuals (10 females; age range = 18–34; mean age = 23; ethnicity = 64% White, 18% Hispanic/Latino, 18% Asian) participated in a 1.5-hour learning session immediately followed by a 1.5-hour fMRI scanning session. Behavioral data from a total of 31 individuals were collected, but 7 subjects did not meet the learning criteria from the behavioral task, one subject was unable to be scanned, and one subject’s fMRI data were incomplete. All participants were right-handed (self-reported) with normal or corrected-to-normal vision. Participants provided written informed consent in accordance with the Declaration of Helsinki and the Human Subjects Review Board at George Mason University and were compensated for their time.

### Experimental Design and Statistical Analysis

#### Stimuli.

Task stimuli consisted of 24 faces of varying ethnicities, equally divided by gender. Faces were all in color and facial expressions were all smiling. These stimuli were downloaded from the Park Aging Mind Laboratory Database at UT Dallas (Minear & Park, 2004) and were chosen to be as realistic to a college campus as possible, ensuring the perception of real people who might interact and be friends with each other.

#### Task Design.

**Figure 1.**
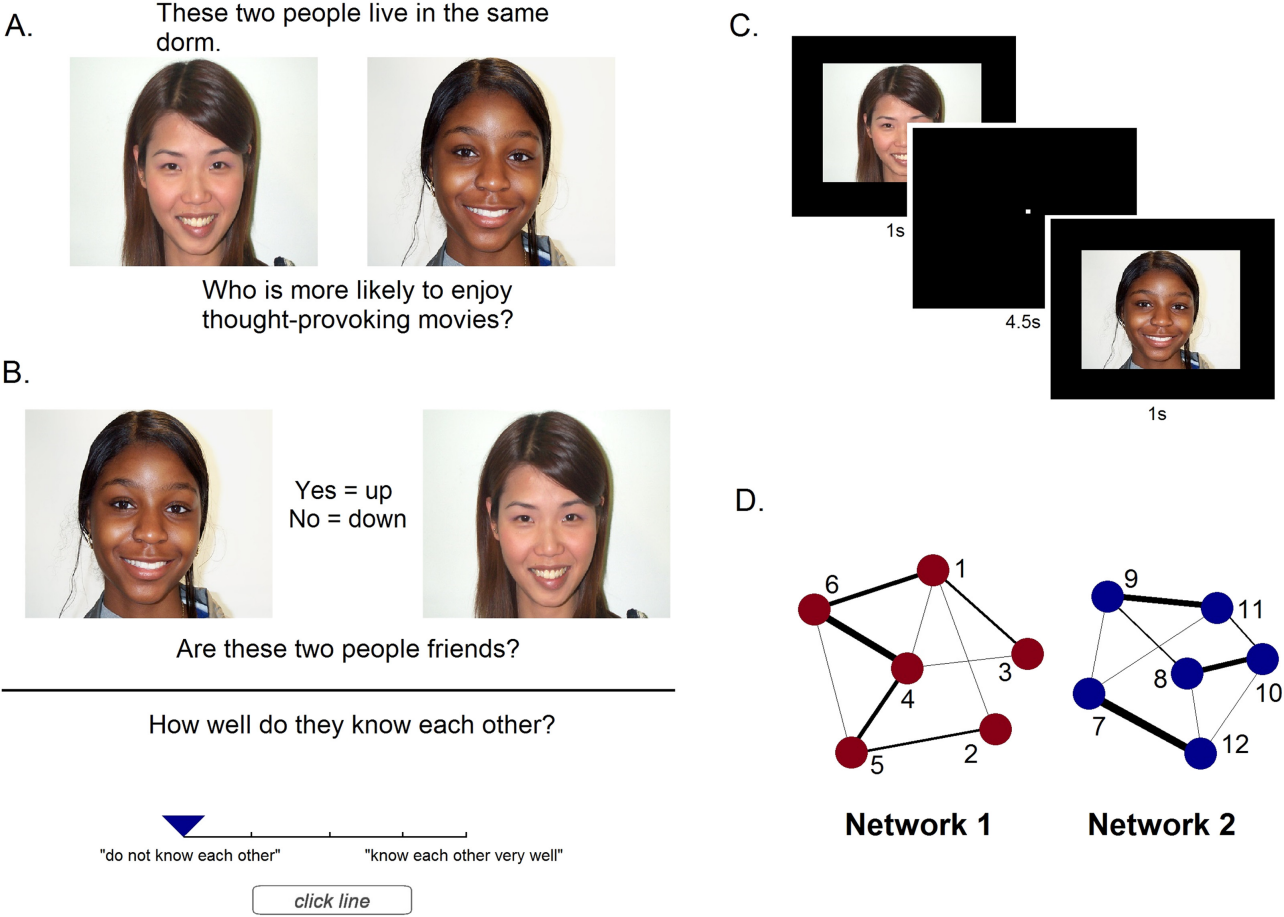
**Details of the experimental design.** A. Example trial of the paired presentation of a social network tie, where subjects were asked to judge between the two faces on an unrelated characteristic. B. Example trial of the recall task, where subjects were asked to report whether a pair of faces was connected, and how well they know each other (0–4 scale). C. fMRI task, where each face was presented individually for 1 second (4.5-second inter-stimulus interval). D. Structure of the two social networks. Each node represents a different face and line thickness represents connection strength. All ties are nondirected (reciprocal).

Participants completed a two-alternative forced-choice task to become familiar with the structure of two 6-person social networks (Figure 1). Pairs of faces represented connections within each network, with the frequency of pairing indicating relationship strength. Each network had an equal number of male and female faces of varying ethnicities. Network properties differed between the two in that although each network had an equal number of connections of each strength level, there were differences among the individual members (faces) in each network. The faces in network 1 had varying numbers of connections and therefore each had a different average closeness to the rest of the network, whereas the faces in network 2 had an equal number of connections and an equal average closeness to all other faces in the network. This meant that in network 1 the centrality of members was varied (variable-centrality network), while in network 2 centrality was equated across members (fixed-centrality network). This also meant that the frequency of presentation of each individual face differed in network 1, but was equivalent in network 2. Each trial consisted of a face pair presented for 4 seconds accompanied by a question, and participants were asked to make a comparison between the faces and decide which person better fit the question. Questions consisted of behavioral and personality characteristics taken from various personality surveys included in the International Personality Item Pool (http://ipip.ori.org/). Half of the questions asked which person was more likely to exhibit a characteristic, and half asked which person was less likely (example: “Who is more likely to be easily intimidated?”). Network learning took place in alternating blocks, where the subjects viewed 36 randomly presented trials of one network followed by 36 trials of the second network. Participants completed 720 trials in total (360 per network), with the weakest network connections being presented a total of 20 times and the strongest a total of 80 times.

After completing the paired face viewing portion, participants were explicitly tested on their knowledge of the network connections. They were told that the faces represented college students living in a dorm together, the faces that they saw paired together previously represented friend connections, and the more often they were presented together, the closer in friendship the pair was. They were asked to group all faces into two separate halls to ensure that they could distinguish between the faces in different networks. They were then presented with all possible within-network face pairs twice and asked to rate their relationship on a scale of 0 (do not know each other) to 4 (know each other very well). They were not asked about cross-network face relationships. This explicit testing period was included to ensure that participants learned the structure of the networks to an appropriate level before being scanned. Participants who were within 2 standard deviations of pilot data (hit rate = .85, *SD* = 0.14; false alarm rate = .35, *SD* = 0.15) were included in further analysis. Both parts of the behavioral task (learning and recall) were presented to the participant using PsychoPy version 1.842 software (Peirce, 2007).

The fMRI task stimuli included the same 12 faces from the behavioral task as well as 12 novel faces as a control. Faces were presented one at a time for one second on a black background with a 4.5-second inter-stimulus interval (black screen with a white fixation dot), and participants completed a 1-back task to ensure attention. The task consisted of four runs of 9.6 minutes each, resulting in each face being presented a total of 16 times (not counting repeats, which were included in analysis as a separate regressor). Following the face task, participants underwent a dynamic localizer session. Localizer stimuli consisted of 18-s blocks each of moving faces, body parts, outdoor scenes, objects, and scrambled objects. The fMRI experiment was presented to the participant using Presentation software (Version 16.3, Neurobehavioral Systems, Inc., Berkeley, CA, www.neurobs.com). Analysis of social network recall data was conducted in Microsoft Excel (version 2016) and R Version 3.3.2 (R Core Team, 2017).

#### Representational Similarity Analysis.

**Figure 2.**
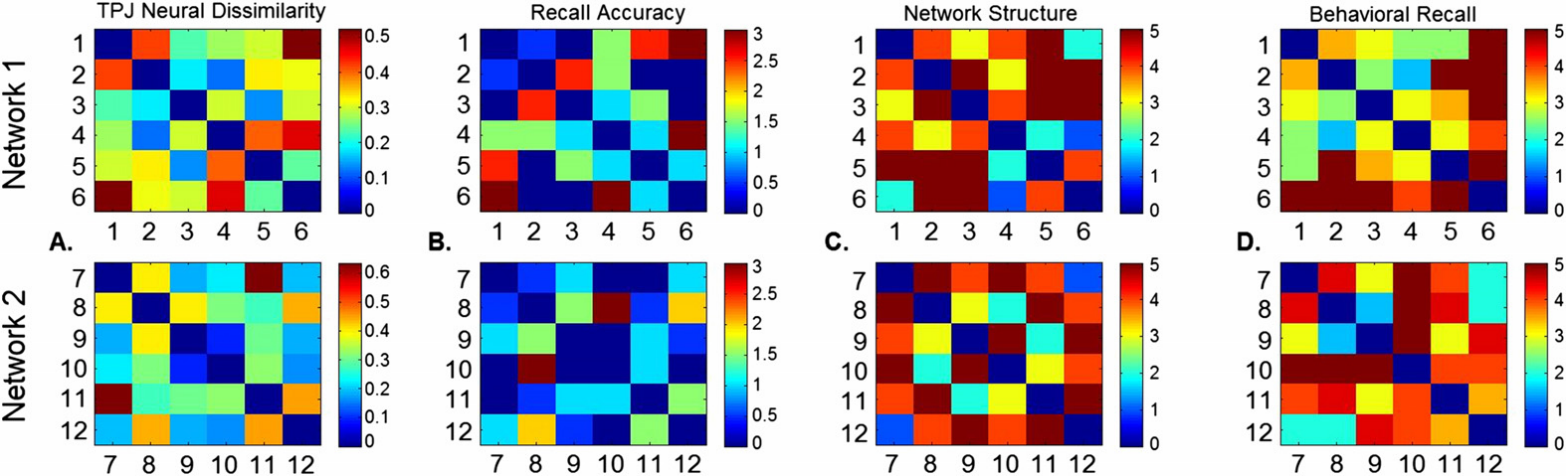
**Dissimilarity matrices between face pairs for a sample subject.** A. Neural dissimilarity in an example region in the temporoparietal junction. B. Recall accuracy DM (0 = perfectly accurate recall). C. True network structure DM (5 = unconnected). D. Behavioral recall of face pair strength (5 = unconnected).

RSA is a form of multivariate pattern analysis that compares the distance between stimuli in neural representational space (Kriegeskorte, Mur, & Bandettini, 2008), and correlates these neural patterns of information with external patterns of information. In this way it can be utilized to assess different models or patterns of cognition above and beyond univariate analysis, or even more traditional multivariate pattern classification techniques (Haxby, Connolly, & Guntupalli, 2014). Four separate dissimilarity matrices (DMs) were created for each network (Figure 2): true network structure, perception of network structure, group average of perceived structure, and recall accuracy. To contrast within-network and cross-network pairs, a DM was created with all 12 faces included. This treated all connected faces the same, then coded within-network unconnected faces as more dissimilar, followed by cross-network unconnected faces. For more detail on this analysis, as well as fMRI preprocessing and univariate analysis information, see the Supplemental Materials (Dziura & Thompson, 2018).

## RESULTS

### Behavioral Task

**Figure 3.**
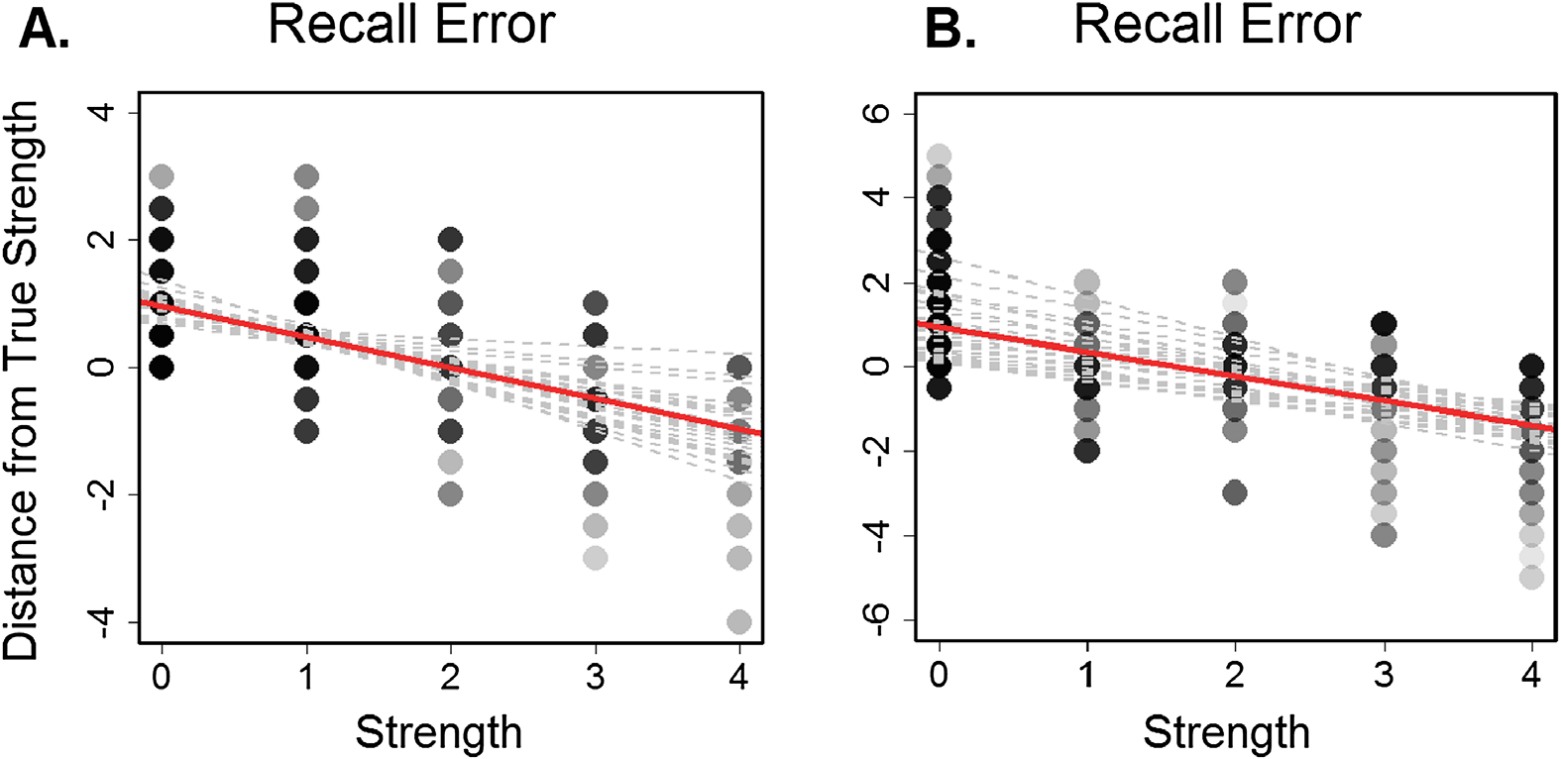
**Subject recall accuracy by relationship strength.** A. Each subject’s error by pair strength level (0 = unconnected, 4 = close friends) from the primary dataset. B. Subject error by pair strength level (0 = unconnected, 6 = close friends) from the secondary dataset with a larger social network (*N* = 9). Positive values = overestimation of strength and negative values = underestimation of strength. Darker colors indicate more data overlap at that point. Gray lines show individual subject regression lines. The red line shows the group regression line.

Subjects correctly identified social network relationship ties significantly greater than chance across both networks [(*t*(21) = 8.08, *p* < .0005]. Table 1 shows the average hit rate, false alarm rate, sensitivity (d′), and the correlation between true and reported perceived strength for ties and relationship strength across subjects. Paired sample two-tailed *t* tests revealed no significant differences between recall measures for the two networks. There were also no significant age or gender effects for any of the measures. When averaged together across subjects, group perceived relationship strength was highly correlated with the true network structure (*r* = .896, *p* < .0005). In order to assess whether our behavioral task was comparable to previous forms of social network learning and recall, we calculated performance measures used by Brashears (2013). Accuracy refers to the number of ties correctly recalled divided by the number of total ties reported, coverage refers to the number of ties correctly recalled divided by the total tie number in the network, and performance refers to the product of accuracy and coverage. *T* tests revealed no significant differences between accuracy or performance measures in our task and those of Brashears [accuracy: *t*(21) = 0.98, *p* = .34; performance: *t*(21) = 0.58, *p* = .56], and we actually saw an increase in coverage [*t*(21) = 3.58, *p* < .005], although our networks were smaller, so participants did not need to remember as many ties. In order to thoroughly explore network recall, we not only looked at the correctly identified ties, but also at any systematic biases that could be predicted by the level of relationship strength of the friend pairs. We assessed recall by relationship strength by the relative direction of the errors made (i.e., how much subjects overestimated or underestimated the strength of the connection). A linear mixed-effects regression model (fixed effect = strength; random effects = subject, residual) revealed that relationship strength affected recall error compared to a null model [χ^2^(1) = 226.9, *p* < .0005]. This pattern shows that overall, weak ties were reported to be stronger than they actually were whereas strong ties were reported to be less strong (Figure 3a). This reflects a general tendency to assume a mid-level relationship between observed people when the relationship is unable to be recalled. This central tendency effect seems to be robust, as it was also observed in a separate subject sample (*N* = 23, 17 females, mean age = 19.6 [*SD* = 2.4]) learning a larger social network (*N* = 9) and a larger possible range of relationship tie strengths to choose from (0–6) (χ^2^(1) = 362.84, *p* < .0005) (Figure 3b). To compare network memory performance to the neural patterns in response to each face in the network, we converted the relative error for each subject to absolute error, which gives a measure of distance from the true network structure, regardless of the direction. The absolute error measure for each subject for each network was then used as a dissimilarity model for RSA to elucidate what neural patterns underlie these memory patterns.

**Table 1.** Accuracy of recalling network relationships after incidental learning.

	**Network 1**	**Network 2**	**Both Networks**	***t* value (*p*)**
**Hit Rate**	**.82**	**.83**	**.83**	**−0.13 (.89)**
*SD*	*0.14*	*0.11*	*0.09*	
**False Alarm Rate**	**.39**	**.47**	**.43**	**−1.23 (.23)**
*SD*	*0.21*	*0.24*	*0.19*	
**d′**	**1.3**	**1.2**	**1.2**	**0.70 (.49)**
*SD*	*0.71*	*0.86*	*0.6*	
**Strength Correlation (*r*)**	**.58**	**.53**	**.54**	**0.82 (.42)**
*SD*	*0.25*	*0.21*	*0.21*	

*Notes*. Hit rate, false alarm rate, and d′ represent the accuracy of recalling the true connections within the networks. Strength correlation refers to the correlation between the matrix of true relationship strength of the faces in the networks and the behavioral judgments of strength, and is therefore a measure of accuracy of recalling relationship strength. *T* values and *p* values for paired sample two-tailed *t* tests between the two networks are reported at the right of the table. Bold indicates primary data, and italics indicate the standard deviation of the data.

### Representational Similarity Analysis

We carried out RSA searchlight analysis on several DMs representing different types of information about the networks. The first compared neural pattern similarity to social tie strength, with more similar neural responses to any pair of faces representing a closer relationship between those faces. Neural pattern similarity that reflects this network structure would indicate that the brain carries information about the true relationship between individuals, regardless of whether people recall those relationships accurately. We did not find a significant correlation between these measures in our analyses. As the network properties differed between networks 1 and 2 (see Methods section for details), we compared the two networks and found no significant differences.

**Figure 4.**
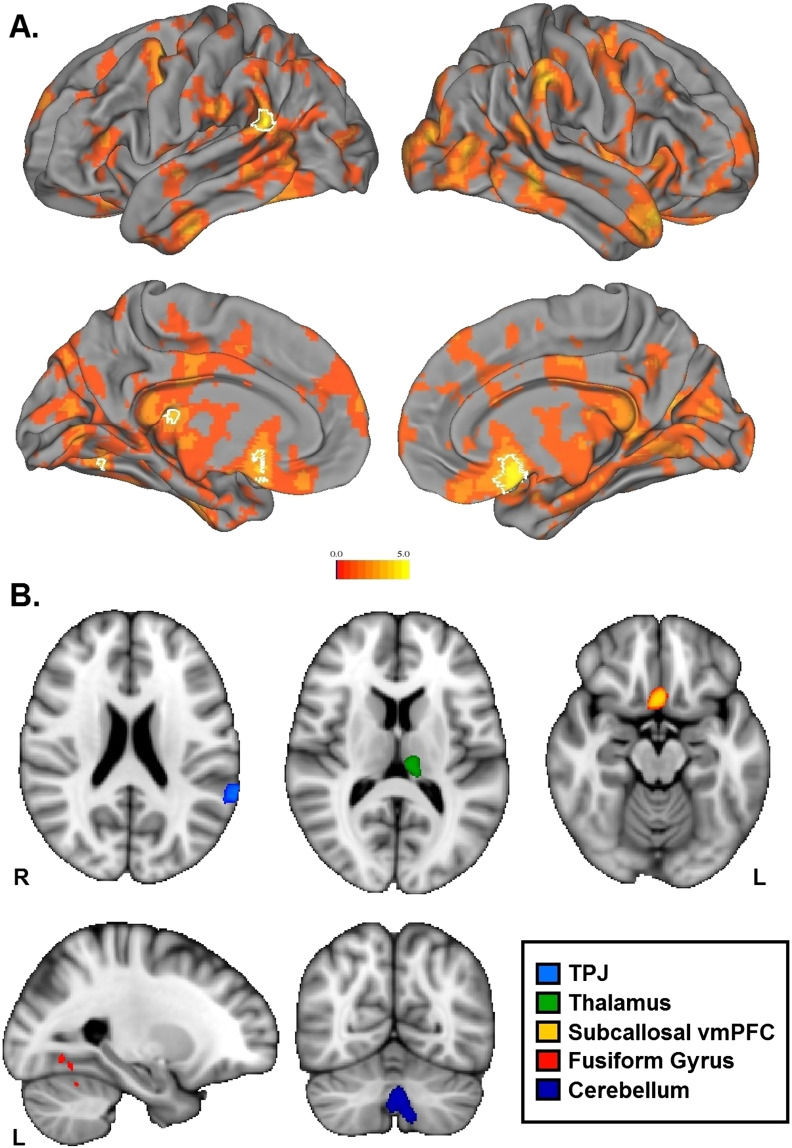
**Results from group-level nonparametric 1-sample *t* test on the correlation maps from RSA searchlight on the recall accuracy model.** A. *t*-statistic map of all positive *t* values projected onto the surface, where white borders delineate significant clusters from the group analysis (*p* < .05, familywise error-corrected with threshold-free cluster enhancement). B. The same significant clusters projected in the volume.

The second RSA searchlight compared neural pattern similarity to the subjects’ memory for network tie strength. We assessed this by measuring each subject’s absolute distance from each true network structure and the 1-correlation distance between the neural response to each face viewed in the scanner. An association between these two measures would indicate that the more accurately a subject perceives the true relationship strength between a pair of faces, the more similar their neural pattern response is to those two faces. In other words, this association does not rely on the actual connection strength of the relationships themselves, but the subject’s memory of that connection, reflecting a second-order knowledge or understanding of a social relationship. Neural pattern similarity in the left TPJ, the left fusiform gyrus, the subcallosal cingulate cortex, the cerebellum, the left thalamus, and a small portion of the left lateral occipital lobe was significantly correlated with the recall accuracy model, suggesting that neural populations within these areas are important for accurate perception of social relationship strength (Figure 4). Table 2 reports MNI coordinates, cluster size, and peak voxel activity of results. As with tie strength similarity, we compared the two networks to each other separately and found no significant differences.

**Table 2.** Coordinates, cluster size, and peak activity for the group-level significant clusters from the recall accuracy model.

**Cluster**	**Peak Value (*t*)**	**Voxels**	***x***	***y***	***z***
Cerebellum	3.92	730	0	−64	−38
Subcallosal vmPFC	5.6	274	2	14	−16
Thalamus	4.14	132	−10	−28	10
TPJ	4.31	117	−64	−48	18
Fusiform Gyrus	3.28	11	−26	−60	−12
Fusiform Gyrus	3.5	5	−24	−66	−8
Fusiform Gyrus	3.37	5	−26	−56	26
Lateral Occipital	3.65	3	−52	−56	2
Lateral Occipital	3.66	3	−56	−70	−2

We also conducted RSA searchlights using two other dissimilarity matrix models: recalled structure as measured by behavioral judgments, and the group average of those behavioral judgments (Figure 4). Neural pattern similarity that reflects behavioral recall would indicate that the brain carries information about an individual’s perception of relationships, regardless of how accurate those perceptions are. However, neither model reached significance. Finally, we utilized a separate functional localizer to create regions of interest selective for face processing in the bilateral superior temporal sulcus (STS) and fusiform gyrus. We also created bilateral hippocampus regions of interest (ROI) using anatomical subcortical segmentation. We conducted RSA correlations across each ROI for every subject (see the Supplemental Materials [Dziura & Thompson, 2018]). No regions yielded significant results.

## DISCUSSION

In this study, we used fMRI and RSA to examine the neural representational space of social connections, learned from observing how frequently individuals were paired together. We examined if either (1) the tie strength between pairs of network members or (2) the memory for the relationship between pairs of network members was represented in the human brain via the similarity of fMRI responses associated with viewing the faces of those members. We found support for the second proposal, but not the first. The accuracy of relationship recall was represented in several brain regions, including the TPJ, subcallosal vmPFC, fusiform gyrus, cerebellum, and thalamus. Our results indicate that these regions represent memory or knowledge of the association between pairs of faces. That is, these areas code memory for association strength regardless of what that strength is. The more accurately a participant recalled the tie strength for a pair of faces, the more similar the pattern of fMRI responses was to viewing those two faces.

The correlation between neural pattern similarity and knowledge of a relationship between network members is a second-order association. Previous studies have shown that this is not the sole mechanism for representing knowledge about relationships between people; neural pattern similarity also codes direct social distance in other brain areas (Parkinson et al., 2017). It is possible that the incidental learning task did not allow for these social network relationships to be embedded enough to see this relationship in all our subjects, unlike real-world or personal relationships that hold more importance to individuals. However, it may be useful to represent this second-order knowledge about social information as well, especially during the process of learning new relationships, or when gathering information about the social world. Confidence in our knowledge about others can affect how we interact with them. In simple terms, we have shown that the brain not only codes what we know, but also how well we know it.

Both the TPJ and vmPFC have been consistently linked with knowledge and recall of complex social information, such as tracking the popularity of real-world social network members (Zerubavel et al., 2015). The TPJ, dorsomedial PFC, and ventrolateral PFC are engaged when participants recall different facets of socially relevant knowledge (Satpute et al., 2014). The left TPJ is selectively modulated by vasopressin, a neuropeptide linked to complex social behaviors, during social recognition (Zink et al., 2011) and lesions to the left TPJ lead to specific deficits in social reasoning (Samson, Apperly, Chiavarino, & Humphreys, 2004). The vmPFC shows increased activation when thinking about friends compared to kin (Wlodarski & Dunbar, 2016), and the subgenual cingulate cortex is involved in tracking individual differences in perceptions of cohesiveness in kin groups (Rüsch et al., 2014). Our findings are in line with this previous literature showing the importance of these areas in forming and maintaining social relationships. The fusiform gyrus is also involved in social perception, particularly in response to face stimuli (Atkinson & Adolphs, 2011; Kanwisher, McDermott, & Chun, 1997). Our data indicate that patches of the fusiform gyrus do not simply perceive and distinguish facial features, but are also involved in learning more abstract relationships between faces. Areas of the cerebellum are also consistently activated in several features of social cognition, with increases in activity occurring with increasing social abstraction levels in the cognitive tasks (Overwalle, Baetens, Marien, & Vandekerckhove, 2014). Our finding that the cerebellum is involved in accurate knowledge of abstract learned relationships between others is consistent with this. Furthermore, we found that the thalamus is also involved in this process. The thalamus has a large number of connections to other areas of the brain, and has been shown to have specific emotional and socially relevant associations (Christoffel et al., 2015; Feng et al., 2016; Ioannidis et al., 2013). It also has high functional connectivity to the hippocampus (Stein et al., 2000), and may be a critical link in the formation of episodic memories (Aggleton et al., 2010).

While most of our subjects were able to accurately report relationship ties, there were individual differences between ability to recall relationship strength, consistent with prior literature (Casciaro, 1998). People also show differences in the ability to perceive and remember nonsocial patterns, but evidence suggests that learning, remembering, and storing social information might be distinct from traditional learning and memory systems (Meyer, Taylor, & Lieberman, 2015; Okuyama, Kitamura, Roy, Itohara, & Tonegawa, 2016; Tendler & Wagner, 2015). In this study we examined the representation of social connections acquired incidentally from the frequency of co-occurrence of different face pairs. While such paired associates-type learning has its limitations in modeling the rich and complex nature of social connections acquired in real life, it is likely that we learn the statistics of connections between people in part through repeated observation of their association. This study does not directly examine whether these associative neural patterns are exclusively social, as all learned pairs were faces within a larger network. However, unlike previous studies of the representation of statistical regularities of abstract, nonsocial stimuli, we did not observe representation in medial temporal regions (Garvert, Dolan, & Behrens, 2017; Schapiro, Kustner, & Turk-Browne, 2012; Schapiro, Turk-Browne, Norman, & Botvinick, 2016). Future work might seek to determine the extent to which memory for social connections reflects the involvement of a general purpose system or if there are distinct mechanisms for representing this kind of social information.

The way in which people learn and remember associations between individuals in groups has a considerable impact on everyday life. We are not only able to perceive and understand the social signals of other individuals, but we can also perceive and understand information about social connections or relationships in which we are not directly involved. Our results show that the memory for these indirect connections is coded in the pattern of neural responses associated with viewing related individuals. This is an important skill because the accuracy with which we see and remember subtle associations from our surroundings helps us move more freely and easily in our highly social world.

## FUNDING INFORMATION

JCT, Office of Naval Research (http://dx.doi.org/10.13039/100000006), Award ID: N00014-10-1-0198.

## AUTHOR CONTRIBUTIONS

SLD: Data curation: Lead; Formal analysis: Lead; Investigation: Lead; Methodology: Equal; Validation: Lead; Visualization: Lead; Writing – original draft: Lead; Writing – review & editing: Equal. JCT: Conceptualization: Lead; Formal analysis: Supporting; Funding acquisition: Lead; Methodology: Equal; Project administration: Lead; Supervision: Lead; Writing – original draft: Supporting; Writing – review & editing: Equal.

## References

[bib1] Aggleton, J. P., O’Mara, S. M., Vann, S. D., Wright, N. F., Tsanov, M., & Erichsen, J. T. (2010). Hippocampal-anterior thalamic pathways for memory: Uncovering a network of direct and indirect actions. The European Journal of Neuroscience, 31, 2292–2307. 20550571 10.1111/j.1460-9568.2010.07251.x PMC2936113

[bib2] Atkinson, A. P., & Adolphs, R. (2011). The neuropsychology of face perception: Beyond simple dissociations and functional selectivity. Philosophical Transactions of the Royal Society B, 366, 1726–1738. 10.1098/rstb.2010.0349 PMC3130374 21536556

[bib3] Bault, N., Pelloux, B., Fahrenfort, J. J., Ridderinkhof, K. R., & van Winden, F. (2015). Neural dynamics of social tie formation in economic decision-making. Social Cognitive and Affective Neuroscience, 10, 877–884. 25338630 10.1093/scan/nsu138 PMC4448033

[bib4] Brashears, M. E. (2013). Humans use compression heuristics to improve the recall of social networks. Scientific Reports, 3, 1–7. 10.1038/srep01513 PMC3604710 23515066

[bib5] Brent, L. J. (2015). Friends of friends: Are indirect connections in social networks important to animal behavior? Animal Behaviour, 103, 211–222. 25937639 10.1016/j.anbehav.2015.01.020 PMC4415378

[bib6] Casciaro, T. (1998). Seeing things clearly: Social structure, personality, and accuracy in social network perception. Social Networks, 20, 331–351.

[bib7] Cheney, D. L. (2011). Extent and limits of cooperation in animals. Proceedings of the National Academy of Sciences, 108, 10902–10909. 10.1073/pnas.1100291108 PMC3131815 21690405

[bib8] Christoffel, D. J., Golden, S. A., Walsh, J. J., Guise, K. G., Heshmati, M., Friedman, A. K., … Russo, S. J. (2015). Excitatory transmission at thalamo-striatal synapses mediates susceptibility to social stress. Nature Neuroscience, 18, 962–964. 26030846 10.1038/nn.4034 PMC4482771

[bib9] De Soto, C. B. (1960). Learning a social structure. Journal of Abnormal and Social Psychology, 60, 417–421. 13815066 10.1037/h0047511

[bib10] De Soto, C. B., & Bosley, J. J. (1962). The cognitive structure of a social structure. Journal of Abnormal and Social Psychology, 64, 303–307. 13884554 10.1037/h0044867

[bib11] Dziura, S. L., & Thompson, J. C. (2018). Supplemental material for “the neural representational space of social memory.” Open Mind: Discoveries in Cognitive Science, 3, 1–12. 10.1162/opmi_a_00021 PMC8412184 34485787

[bib12] Feng, C., Li, Z., Feng, X., Wang, L., Tian, T., & Luo, Y. J. (2016). Social hierarchy modulates neural responses of empathy for pain. Social Cognitive and Affective Neuroscience, 11, 485–495. 26516169 10.1093/scan/nsv135 PMC4769637

[bib13] Freeman, L. C., Freeman, S. C., & Michaelson, A. G. (1988). On human social intelligence. Journal of Social and Biological Structures, 11, 415–425.

[bib14] Garvert, M. M., Dolan, R. J., & Behrens, T. E. J. (2017). A map of abstract relational knowledge in the human hippocampal-entorhinal cortex. eLife, 6, e17086. 28448253 10.7554/eLife.17086 PMC5407855

[bib15] Haxby, J. V., Connolly, A. C., & Guntupalli, J. S. (2014). Decoding neural representational spaces using multivariate pattern analysis. Annual Reviews of Neuroscience, 37, 435–456. 10.1146/annurev-neuro-062012-170325 25002277

[bib16] Ioannidis, A. E., Kimiskidis, V. K., Loukopoulou, E., Geroukis, T., Vlaikidis, N., & Kosmidis, M. H. (2013). Apathy, cognitive dysfunction and impaired social cognition in a patient with bilateral thalamic infarction. Neurocase, 19, 513–520. 22816313 10.1080/13554794.2012.701645

[bib17] Janicik, G. A., & Larrick, R. P. (2005). Social network schemas and the learning of incomplete networks. Journal of Personality and Social Psychology, 88, 348–364. 15841863 10.1037/0022-3514.88.2.348

[bib18] Kanwisher, N., McDermott, J., & Chun, M. M. (1997). The fusiform face area: A module in human extrastriate cortex specialized for face perception. The Journal of Neuroscience, 17, 4302–4311. 9151747 10.1523/JNEUROSCI.17-11-04302.1997 PMC6573547

[bib19] Kriegeskorte, N., Mur, M., & Bandettini, P. (2008). Representational similarity analysis—Connecting the branches of systems neuroscience. Frontiers in Systems Neuroscience, 2, 1–28. 19104670 10.3389/neuro.06.004.2008 PMC2605405

[bib20] Kumaran, D., Melo, H. L., & Duzel, E. (2012). The emergence and representation of knowledge about social and nonsocial hierarchies. Neuron, 76, 653–666. 23141075 10.1016/j.neuron.2012.09.035 PMC3580285

[bib21] Leavitt, H. J. (1951). Some effects of certain communication patterns on group performance. The Journal of Abnormal and Social Psychology, 46, 38–50. 10.1037/h0057189 14813886

[bib22] Lin, N., Dayton, P. W., & Greenwald, P. (1978). Analyzing the instrumental use of relations in the context of social structure. Sociological Methods & Research, 7, 149–166.

[bib23] Meyer, M. L., Taylor, S. E., & Lieberman, M. D. (2015). Social working memory and its distinctive link to social cognitive ability: An fMRI study. Social Cognitive and Affective Neuroscience, 10, 1338–1347. 25987597 10.1093/scan/nsv065 PMC4590544

[bib24] Minear, M., & Park, D. C. (2004). A lifespan database of adult facial stimuli. Behavior Research Methods, Instruments, & Computers, 36, 630–633. 10.3758/bf03206543 15641408

[bib25] Okuyama, T., Kitamura, T., Roy, D. S., Itohara, S., & Tonegawa, S. (2016). Ventral CA1 neurons store social memory. Science, 353, 1536–1541. 27708103 10.1126/science.aaf7003 PMC5493325

[bib26] Overwalle, F. V., Baetens, K., Marien, P., & Vandekerckhove, M. (2014). Social cognition and the cerebellum: A meta-analysis of over 350 fMRI studies. NeuroImage, 86, 554–572. 24076206 10.1016/j.neuroimage.2013.09.033

[bib27] Parkinson, C., Kleinbaum, A. M., & Wheatley, T. (2017). Spontaneous neural encoding of social network position. Nature Human Behaviour, 1, 1–7.

[bib28] Peirce, J. W. (2007). PsychoPy-Psychophysics software in Python. Journal of Neuroscience Methods, 162, 8–13. 17254636 10.1016/j.jneumeth.2006.11.017 PMC2018741

[bib29] Rüsch, N., Bado, P., Zahn, R., Bramati, I. E., De Oliveira-Souza, R., & Moll, J. (2014). You and your kin: Neural signatures of family-based group perception in the subgenual cortex. Social Neuroscience, 9, 326–331. 24802255 10.1080/17470919.2014.912676 PMC4047618

[bib30] Samson, D., Apperly, I. A., Chiavarino, C., & Humphreys, G. W. (2004). Left temporoparietal junction is necessary for representing someone else’s belief. Nature Neuroscience, 7, 499–500. 15077111 10.1038/nn1223

[bib31] Satpute, A. B., Badre, D., & Ochsner, K. N. (2014). Distinct regions of prefrontal cortex are associated with the controlled retrieval and selection of social information. Cerebral Cortex, 24, 1269–1277. 23300111 10.1093/cercor/bhs408 PMC3977620

[bib32] Schapiro, A. C., Kustner, L. V., & Turk-Browne, N. B. (2012). Shaping of object representations in the human medial temporal lobe based on temporal regularities. Current Biology, 22, 1622–1627. 22885059 10.1016/j.cub.2012.06.056 PMC3443305

[bib33] Schapiro, A. C., Turk-Browne, N. B., Norman, K. A., & Botvinick, M. M. (2016). Statistical learning of temporal community structure in the hippocampus. Hippocampus, 26, 3–8. 26332666 10.1002/hipo.22523 PMC4715493

[bib34] Seyfarth, R. M., & Cheney, D. L. (2012). The evolutionary origins of friendship. Annual Review of Psychology, 63, 153–177. 10.1146/annurev-psych-120710-100337 21740224

[bib35] Seyfarth, R. M., & Cheney, D. L. (2015). Social cognition. Animal Behaviour, 103, 191–202.

[bib36] Stein, T., Moritz, C., Quigley, M., Cordes, D., Haughton, V., & Meyerand, E. (2000). Functional connectivity in the thalamus and hippocampus studied with functional MR imaging. American Journal of Neuroradiology, 21, 1397–1401. 11003270 PMC7974059

[bib37] Tendler, A., & Wagner, S. (2015). Different types of theta rhythmicity are induced by social and fearful stimuli in a network associated with social memory. eLife, 4, e03614. 10.7554/eLife.03614 PMC4353977 25686218

[bib38] Tiddi, B., Sorrentino, E. P. D., Fischer, J., & Schino, G. (2017). Acquisition and functional consequences of social knowledge in macaques. Royal Society Open Science, 4, 160639. 28386423 10.1098/rsos.160639 PMC5367287

[bib39] Tung, J., Barreiro, L. B., Burns, M. B., Grenier, J. C., Lynch, J., Greineisen, L. E., … Archie, E. A. (2015). Social networks predict gut microbiome composition in wild baboons. eLife, 4, e05224. 10.7554/eLife.05224 PMC4379495 25774601

[bib40] Turk-Browne, N. B., Scholl, B. J., Johnson, M. K., & Chun, M. M. (2010). Implicit perceptual anticipation triggered by statistical learning. The Journal of Neuroscience, 30, 11177–11187. 20720125 10.1523/JNEUROSCI.0858-10.2010 PMC2947492

[bib41] Wlodarski, R., & Dunbar, R. I. M. (2016). When BOLD is thicker than water: Processing social information about kin and friends at different levels of the social network. Social Cognitive and Affective Neuroscience, 11, 1952–1960. 27576746 10.1093/scan/nsw101 PMC5141953

[bib42] Zerubavel, N., Bearman, P. S., Weber, J., & Ochsner, K. N. (2015). Neural mechanisms tracking popularity in real-world social networks. Proceedings of the National Academy of Sciences, 112, 15072–15077. 10.1073/pnas.1511477112 PMC4679039 26598684

[bib43] Zink, C. F., Kempf, L., Hakimi, S., Rainey, C. A., Stein, J. L., & Meyer-Lindenberg, A. (2011). Vasopressin modulates social recognition-related activity in the left temporoparietal junction in humans. Translational Psychiatry, 1, e3. 22832391 10.1038/tp.2011.2 PMC3309468

[bib44] Zink, C. F., Tong, Y., Chen, Q., Bassett, D. S., Stein, J. L., & Meyer-Lindenberg, A. (2008). Know your place: Neural processing of social hierarchy in humans. Neuron, 58, 273–283. 18439411 10.1016/j.neuron.2008.01.025 PMC2430590

[bib45] Zitek, E. M., & Tiedens, L. Z. (2012). The fluency of social hierarchy: The ease with which hierarchical relationships are seen, remembered, learned, and liked. Journal of Personality and Social Psychology, 102, 98–115. 21910553 10.1037/a0025345

