# Supplementary material for: The Neural Representational Space of Social Memory
Source: Open Mind (Camb). 2019 Feb 1;3:1–12. doi: 10.1162/opmi_a_00021 (PMC8412184; doi:10.1162/opmi_a_00021)
Supplement: Supplementary file 1 [file opmi-03-1-s001.pdf]

# Supplementary Methods

## fMRI data acquisition, preprocessing, and analysis

fMRI data were collected with a Siemens Allegra 3T scanner and a quadrature birdcage head coil at the Department of Psychology at George Mason University. Visual stimuli were displayed on a rear projection screen and viewed by participants on a head coil-mounted mirror. Blood oxygenation level dependent (BOLD) data were acquired using gradient-echo, echoplanar imaging scans: 40 axial slices (3-mm slice thickness), repetition time (TR)/echo time (TE) = 2350/30 ms, flip angle = 70, 64 x 64 matrix, field of view = 192 mm. 245 volumes were collected in each run. At the end of the fMRI scanning session, one T1 whole-head anatomical structural scan was acquired using a three-dimensional, magnetization-prepared, rapid-acquisition gradient echo (MPRAGE) pulse sequence. The following parameters were used for these scans: 160 1-mm slices, 256 x 256 matrix, field of view = 260 mm, TR/TE = 2300/3.37 ms. Functional data were analyzed using FSL (version 5.0.8) fMRI Expert Analysis Tool (fsl.fmrib.ox.ac.uk) and Matlab (version R2012a) software (http://www.mathworks.com). Preprocessing included BET non-brain removal, high-pass temporal filtering at 96 s, slice-timing correction, motion correction, and smoothing with a 4 mm FWHM kernel. Runs with > 1 mm of motion were run through the BrainWavelet Despiking program in Matlab (Patel et al., 2014). For first-level analysis, linear regression was conducted at each voxel, using generalized least squares with a voxel-wise, temporally and spatially regularized autocorrelation model, drift fit with Gaussian-weighted running line smoother. For second-level analysis, linear regression was conducted at each voxel, using ordinary least squares.

**fMRI Univariate Analysis**

Each subject's functional task data was registered to his or her anatomical scan and then registered to the MNI standard template. Unlike processing for the RSA analysis, this task data was smoothed with a 6 mm FWHM kernel during preprocessing. The regressors used in the generalized linear modeling (GLM) analysis were Network 1, Network 2, Control, and Response Trials. Contrasts used were Network 1 v. Control, Network 2 v. Control, Both Networks v. Control, Control v. Both Networks, Network 1 v. Network 2, and Network 2 v. Network 1. Group nonparametric 1-sample (regressors) and 2-sample (contrasts) t-tests (5000 permutations) were conducted with fslrandomise (Winkler, Ridgway, Webster, Smith, & Nichols, 2014) within the mask created from anatomically-defined regions selective for face processing and memory. These tests included threshold-free cluster enhancement to boost signal sensitivity (Smith & Nichols, 2009) and variance smoothing with a sigma of 8 mm to reduce spatial frequency noise in the statistic image (Holmes, Blair, Watson, & Ford, 1996).

**Regions of Interest (ROI) and Mask Creation**

Localizer functional data was registered to each subject's specific structural image. Face-selective regions of interest (ROIs) were created from subtracting the combined object, scrambled object, and scene conditions from the face condition. These regions included bilateral posterior STS and fusiform face area (FFA). Activity was thresholded at $Z > 3.7$ ($p < 0.0001$) for most ROIs, although this threshold was relaxed to $Z > 3$ ($p < 0.001$) in one subject, $Z > 2.3$ ($p < 0.01$) in four subjects, and $Z > 1.65$ ($p < 0.05$) in three subjects because of lower overall BOLD activity. These masks were projected back into native functional space for further analysis. Bilateral hippocampus ROIs were created from the automatic subcortical segmentation of each

subject's T1-weighted MRI scan in Freesurfer (Fischl et al., 2002). Finally, a group-level standard anatomical mask of areas involved in memory for faces (encompassing the bilateral pSTS, extrastriate body area (EBA), ventral temporal/fusiform gyrus, precuneus/posterior cingulate cortex (PC/PCC), and hippocampus) was created from the Harvard-Oxford Cortical Structural Atlas in FSL.

**Representational Similarity Analysis**

For initial analysis of fMRI task data, no registration to structural brain images were carried out. The GLM included separate regressors for each of the 24 faces and repeats. Resulting z-statistics were grouped by network for further analysis. Four separate dissimilarity matrices (DMs) were created for each network: true network structure (created from tie strength), perception of network structure (taken from each subject's behavioral recall data after learning the networks), group average of perceived structure (where each face pair's perceived strength was averaged across subjects), and recall accuracy (measured by calculating the absolute distance between the true strength of each face pair and the average strength of the pair reported in the recall phase). The CoSMoMVPA toolbox in Matlab was used for RSA calculations (Oosterhof, Connolly, & Haxby, 2016).

Whole-brain searchlights using Spearman correlations (size = 50 voxels) were conducted on the z-statistics for each face averaged across all runs. This analysis compared pairwise dissimilarity of the neural response to each face with each DM created for each social network. The ensuing correlation maps were transformed into standard space for group analysis. No significant group differences were found in the brain across the two networks (in whole-brain group nonparametric paired-sample t-tests with 5000 permutations), so the correlation maps in

individual subject space were then averaged across networks within subjects and transformed again to standard space for across-network group analysis. Group nonparametric 1-sample t-tests (5000 permutations) including threshold-free cluster enhancement and variance smoothing of 8 mm were conducted with fslrandomise. Resulting t-statistic maps were visualized in the MNI volume as well as transformed to the PALS-B12 standard atlas in Caret (http://www.nitrc.org/projects/caret/) for surface data visualization (Van Essen, 2005). RSA was also carried out within individual localizer-defined ROIs and the resulting correlations within each region were averaged across subjects.

## Supplemental Results

### fMRI Univariate Results

During fMRI scanning, participants viewed the original faces from the social network behavioral session, as well as 12 novel faces, and were asked to press a button when they saw a repeated face to guarantee attention. We conducted a GLM comparing the 12 familiar faces from the two networks to unfamiliar control faces. **Supplemental Figure 1** shows that an area of the left fusiform gyrus was more active when viewing unfamiliar faces, whereas the posterior cingulate gyrus/precuneus was more active when viewing familiar faces ($p < 0.05$, FWE-corrected with threshold-free cluster enhancement within an anatomical mask composed of areas previously shown to be relevant for face perception and memory; see **Supplemental Table 1** for cluster information). While perception for different categories of faces is highly dependent on task demands, our findings are consistent with some previous literature examining recognition of familiar faces (Natu & O’Toole, 2011). The fusiform gyrus has been shown to activate significantly less to famous faces than to strangers in the left hemisphere (Gobbini, Leibenluft,

Santiago, & Haxby, 2004), and the posterior cingulate/precuneus area is consistently activated more to personally familiar faces when compared to strangers (Gobbini et al., 2004; Pierce, Haist, Sedaghat, & Courchesne, 2004; Sugiura et al., 2001). There were no univariate differences between responses to faces across the two networks.

**Representational Similarity Analysis**

A DM was created for all 12 learned faces across both networks, to examine possible differences between within-network indirect and cross-network indirect connections. This model coded cross-network unconnected faces as most dissimilar, followed by within-network unconnected faces, followed by within-network connected faces (**Supplemental Figure 2**). The DM was correlated with neural pattern similarity across the whole brain in a searchlight with the same methods as the network-specific analyses, and yielded no significant results, corrected for multiple comparisons.

**Non-monotonic Function Exploratory Analysis**

It is possible that the relationship between neural dissimilarity and social relationship strength is non-linear, and in particular non-monotonic. For example, the pairs of faces that are seen most often together could cause people to learn distinguishing features between the two (as once we know two people well, and we know they are friends, we then have to be able to tell them apart). On the other hand, two people who are paired together less often are associated with each other, but not very strongly. In this example, there would be a certain level of relationship strength in between these two that would lead to the highest association with neural pattern similarity. In this case, the function would be u-shaped, and a correlation between the two

variables would not yield significant results in either the whole-brain or region of interest analysis.

As an exploratory analysis to see whether there is support for this theory, we created spherical ROIs with a diameter of 3.5 mm around the left TPJ and vmPFC for every subject. Neural pattern distance data from each pair of faces for each subject was extracted and all pairs of faces with the same relationship strength were then averaged for each subject. Fitting a nonlinear polynomial function to this data (IV = relationship strength, DV = neural pattern distance) did not yield significant results (TPJ: $\beta 1 = 0.27$, $p = 0.25$; $\beta 2 = 0.24$, $p = 0.29$ / vmPFC: $\beta 1 = -0.10$, $p = 0.6$; $\beta 2 = -0.08$, $p = 0.7$). This suggests that the significant results found in the searchlight analysis can not be explained in this manner.

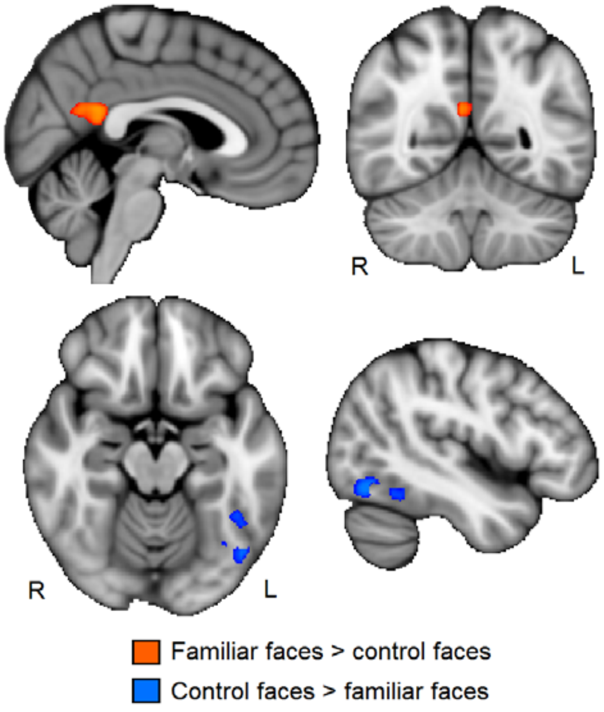

**Supplemental Figure 1**. Results from group analysis of z-statistic maps of familiar (network 1 and 2) faces vs. control faces ($p < 0.05$, FWE-corrected with threshold-free cluster enhancement within an anatomical mask composed of areas previously shown to be relevant for face perception and memory).

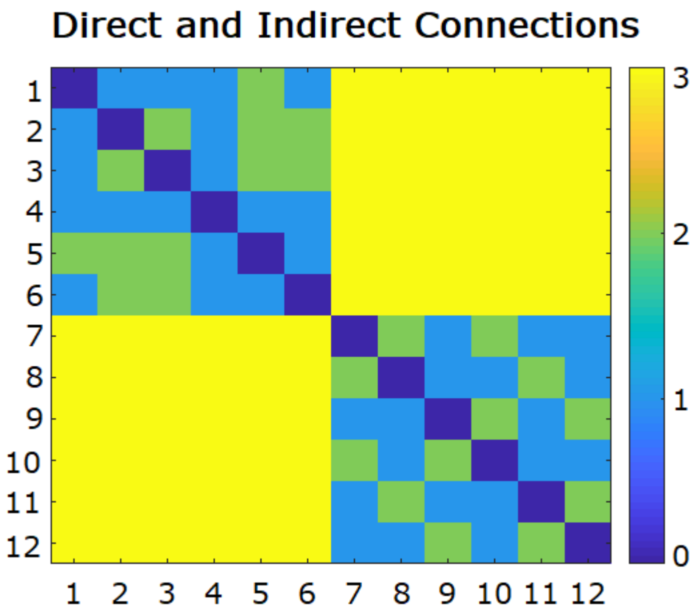


**Supplemental Figure 2**. Dissimilarity matrix between face pairs across both networks. Network 1 = 1-6; Network 2 = 7-12. Similarity structure is as follows: 0 = same face; 1 = connected; 2 = unconnected within the same network; 3 = unconnected in different networks.

| Cluster | Peak Value (t) | Voxels | x | y | z |
|---|---|---|---|---|---|
| **Familiar > Control** | | | | | |
| *Posterior cingulate/precuneus* | 4.44 | 152 | 6 | -48 | 20 |
| *Posterior cingulate/precuneus* | 3.62 | 7 | 2 | -36 | 30 |
| *Posterior cingulate/precuneus* | 3.37 | 6 | -4 | -60 | 32 |
| **Control > Familiar** | | | | | |
| *L fusiform gyrus* | 4.01 | 248 | -44 | -68 | -14 |
| *L fusiform gyrus* | 4.11 | 30 | -38 | -90 | -8 |
| *L fusiform gyrus* | 4.19 | 11 | -24 | -30 | -26 |

**Supplemental Table 1**. Coordinates, cluster size, and peak activity for the group-level clusters from the univariate familiar vs. control face analysis.

**References**

Fischl, B., Salat, D. H., Busa, E., Albert, M., Dieterich, M., Haselgrove, C., Van Der Kouwe, A., Killiany, R., Kennedy, D., Klaveness, S., Montillo, A., Makris, N., Rosen, B., & Dale, A. M. (2002). Whole brain segmentation: Automated labeling of neuroanatomical structures in the human brain. *Neuron, 33,* 341-355.

Gobbini, M. I., Leibenluft, E., Santiago, N., & Haxby, J. V. (2004). Social and emotional attachment in the neural representation of faces. *NeuroImage 22,* 1628-1635. doi: 10.1016/j.neuroimage.2004.03.049.

Holmes, A. P., Blair, R. C., Watson, J. D. G., & Ford, I. (1996). Nonparametric analysis of statistic images from functional mapping experiments. *Journal of Cerebral Blood Flow and Metabolism, 16,* 7-22.

Natu, V. & O'Toole, A. J. (2011). The neural processing of familiar and unfamiliar faces: A review and synopsis. *British Journal of Psychology, 102,* 726-747. doi: 10.1111/j.2044-8295.2011.02053.x.

Oosterhof, N. N., Connolly, A. C., and Haxby, J. V. (2016). CoSMoMVPA: Multi-modal multivariate pattern analysis of neuroimaging data in Matlab / GNU Octave. *Frontiers in Neuroinformatics, 10,* 1-27. doi: 10.3389/fninf.2016.00027.

Patel, A. X., Kundu, P., Rubinov, M., Jones, P. S., Vértes, P. E., Ersche, K. D., Suckling, J., & Bullmore, E. T. (2014). A wavelet method for modeling and despiking motion artifacts from resting-state fMRI time series. *NeuroImage, 95,* 287-304. doi: 10.1016/j.neuroimage.2014.03.012.

Pierce, K., Haist, F., Sedaghat, F., & Courchesne, E. (2004). The brain response to personally familiar faces in autism: Findings of fusiform activity and beyond. *Brain, 127,* 2703–2716. DOI: 10.1093/brain/awh289.

Smith, S. M. & Nichols, T. E. (2009). Threshold-free cluster enhancement: Addressing problems of smoothing, threshold dependence and localization in cluster inference. *NeuroImage, 44,* 83-98.

Sugiura, M., Kawashima, R., Nakamura, K., Sato, N., Nakamura, A., Kato, T., Hatano, K., Schormann, T., Zilles, K., Sato, K., Ito, K., & Fukuda, H. (2001). Activation reduction in anterior temporal cortices during repeated recognition of faces of personal acquaintances. *NeuroImage, 13,* 877–890. doi: 10.1006/nimg.2001.0747.

Van Essen, D. C. (2005). A population-average, landmark- and surface-based (PALS) atlas of human cerebral cortex. *NeuroImage, 28,* 635-662. doi: 10.1016/j.neuroimage.2005.06.058.

Winkler, A. M., Ridgway, G. R., Webster, M. A., Smith, S. M., & Nichols, T. E. (2014). Permutation inference for the general linear model. *NeuroImage, 92,* 381-397.
